# Revisiting the Link between Workplace Support for Families, Family Support, Diet Quality, and Satisfaction with Food-Related Life in the Second Year of the Pandemic

**DOI:** 10.3390/nu16162645

**Published:** 2024-08-10

**Authors:** Berta Schnettler, Andrés Concha-Salgado, Ligia Orellana, Mahia Saracostti, Katherine Beroiza, Héctor Poblete, Germán Lobos, Cristian Adasme-Berríos, María Lapo, Leonor Riquelme-Segura, José A. Sepúlveda

**Affiliations:** 1 Departamento de Producción Agropecuaria, Facultad de Ciencias Agropecuarias y Medioambiente, Universidad de La Frontera, Temuco 4811230, Chile; 2 Scientific and Technological Bioresource Nucleus (BIOREN-UFRO), Universidad de La Frontera, Temuco 4811230, Chile; 3 Centro de Excelencia en Psicología Económica y del Consumo, Universidad de La Frontera, Temuco 4811230, Chile; ligia.orellana@ufrontera.cl (L.O.); k.beroiza01@ufromail.cl (K.B.); hector.poblete@ufrontera.cl (H.P.); jose.sepulveda@ufrontera.cl (J.A.S.); 4 Facultad de Especialidades Empresariales, Universidad Católica de Santiago de Guayaquil, Guayaquil 090308, Ecuador; maria.lapo@cu.ucsg.edu.ec; 5 Departamento de Psicología, Facultad de Educación, Ciencias Sociales y Humanidades, Universidad de La Frontera, Temuco 4811230, Chile; andres.concha@ufrontera.cl; 6 Departamento de Trabajo Social, Facultad de Ciencias Sociales, Universidad de Chile, Santiago 8380453, Chile; mahia.saracostti@uchile.cl; 7 Facultad de Economía y Negocios, Universidad de Talca, Talca 3465548, Chile; globos@utalca.cl; 8 Departamento de Economía y Administración, Facultad de Ciencias Sociales y Económicas, Universidad Católica del Maule, Talca 3530000, Chile; cadasme@ucm.cl; 9 Departamento de Trabajo Social, Facultad de Educación, Ciencias Sociales y Humanidades, Universidad de La Frontera, Temuco 4811230, Chile; leonor.riquelme@ufrontera.cl

**Keywords:** dual-earner parents, adolescents, support, satisfaction with food-related life, diet quality, dyadic analysis

## Abstract

The main objective of this study was to examine the actor and partner effects between Perceived Workplace Support for Families (PWSFs) and family support (PFS), diet quality, and satisfaction with food-related life (SWFoL) in households with both parents working and adolescents, along with the role of the three family members’ diet quality as a mediator. During the second year of the pandemic in Chile, 860 dual-earner parents of different sexes and their adolescent child (average age 13 years, with 50.7% being male) were recruited from two cities. Parents responded to a measure of PWSFs and the Perceived Family Support Scale. Mothers, fathers, and adolescents answered the Adapted Healthy Eating Index (AHEI) and the satisfaction with food-related life Scale. The examination employed the mediation Actor–Partner Interdependence Model and structural equation modeling for the analyses. Results showed that mothers’ PWSFs improved their and their teenage children’s SWFoL, while fathers’ PWSFs only improved their SWFoL. The mothers’ PFS improved their and the fathers’ diet quality while enhancing their SWFoL and the adolescents’ SWFoL. The fathers’ PFS enhanced their and the adolescents’ SWFoL. The mothers’ PFS also indirectly enhanced their and the fathers’ SWFoL via each parent’s diet quality. Each family member’s diet quality was positively related to their SWFoL, while mothers’ diet quality was positively related to the fathers’ SWFoL. These results imply that resources obtained by parents from PFS positively impact the SWFoL of the three family members through different mechanisms. They also highlight the importance of maternal family support for SWFoL during the pandemic.

## 1. Introduction

The COVID-19 pandemic challenged working parents, impacting their professional and personal lives. Throughout the pandemic, there was a significant shift in people’s employment circumstances, with many individuals adapting to remote work without adequate resources [1]. This change profoundly impacted family dynamics as household members found themselves confined to their homes due to lockdown restrictions, resulting in heightened responsibilities for workers, including childcare, homeschooling, and various domestic duties, including food-related chores [2,3]. Consequently, although work-related stress increased, stress related to family demands increased even more [4,5], which may decrease workers’ and their family member’s diet quality. Research has shown that poor dietary habits are prevalent among employees in different economic sectors. Such practices significantly impact employee sick leave and productivity, resulting in significant costs for employers and society [6]. Furthermore, poor diet quality may decrease an employee’s satisfaction with food-related life (e.g., [7,8,9]), defined as an individual’s mental evaluation of their food choices and eating behaviors. This includes planning meals, grocery shopping, preparing meals, eating habits, and managing food waste [10]. Satisfaction with food-related life has been positively linked to satisfaction with life, the cognitive component of subjective well-being. Satisfaction with life can be assessed as a whole or by different life domains such as family, work, and food [7,8].

Throughout the pandemic, to cope with the multiple tasks of work and home (including feeding), employees could avail themselves of diverse resources, e.g., Perceived Workplace Support for Families (PWSFs) and Perceived Family Support (family support henceforth), directly improving their and their family’s satisfaction with family life or reducing parents’ family-related stress [11]. PWSFs, derived from the work environment, is a valuable resource that positively influences parenting responsibilities and seeks to minimize the conflict employees experience between work and family [12]. Family support reflects the family members’ interest in the worker’s job. This form of support includes behaviors and attitudes displayed by family members that encourage, understand, provide attention, and positive regard [13].

According to the Conservation of Resources (COR) theory, support is seen as a valuable resource that can assist in meeting demands [14], such as food-related tasks [15]. Although research involving the relationships between workplace and family support and food-related variables is scant, during the initial year of the pandemic in Chile, a study found that PWSFs improved diet quality for working parents and their adolescent children [15]. The COR theory also underscores that support may improve employees’ satisfaction in different life domains [16,17]. In this regard, a pre-pandemic study showed that one parent’s family support is related to their and the other parent’s satisfaction with food-related life [18]. Nevertheless, while the first study [15] did not evaluate family members’ satisfaction with food-related life, the second study [18] neither assessed parents’ diet quality as a possible mediating variable between family support and satisfaction with food-related life nor explored potential relationships in their children. Hence, there is still a limited understanding of how both types of support affect workers’ and their family members’ diet quality and satisfaction with food-related life. At the same time, no study has assessed the relationship between these variables simultaneously in different family members.

Therefore, utilizing the COR theory as a framework, the study sought to investigate the actor and partner effects between PWSFs and family support, diet quality, and satisfaction with food-related life in households with both parents working and adolescents, along with the role of the three family members’ diet quality as a mediator. Families with adolescent children were chosen for the study because adolescents emotionally support working parents [19]. The data were examined utilizing the mediation Actor–Partner Interdependence Model (APIM) framework [20]. The actor effect refers to results based on an individual’s characteristics. On the other hand, partner effects are outcomes based on the characteristics of the other person in the relationship (crossover) [20].

## 2. Background and Hypotheses

### 2.1. Perceived Workplace Support for Families, Family Support, and Satisfaction with Food-Related Life

The COR theory suggests that people aim to obtain, maintain, and safeguard their resources, shielding them from stressors and amassing additional resources linked to positive well-being outcomes (see [16]). Organizations set up policies and procedures to provide resources to assist their employees with their family and personal circumstances, which in turn may improve their satisfaction in other life domains [12,17].

**Figure 1:**
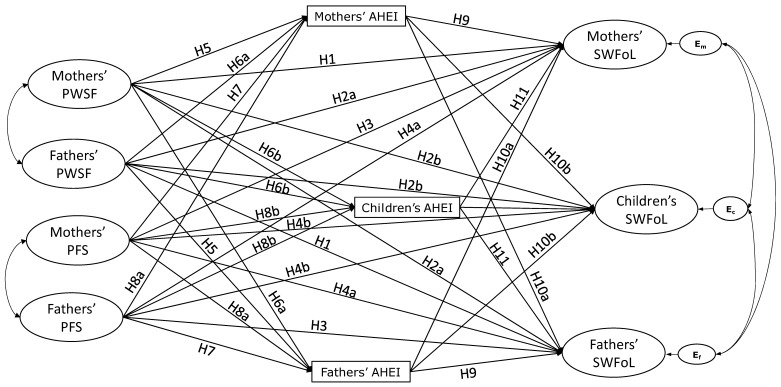
Conceptual model of the proposed actor and partner effects between Perceived Workplace Support for Families (PWSFs), Perceived Family Support (PFS), diet quality (AHEI), and satisfaction with food-related life (SWFoL) in dual-earner parents with teenagers. E_m_, E_c_, and E_f_: residual errors on SWFoL for mothers, teenagers, and fathers, respectively. The indirect effects of diet quality (H12 and 13) were not shown in the conceptual model to avoid cluttering the figure.

In this regard, before the pandemic, a study in Chile revealed that resources parents acquire from the workplace are related to their satisfaction with food-related life [21]. PWSFs is linked to policies promoting a family-friendly work environment that enables workers to manage their daily obligations better [22], which in turn results in higher levels of satisfaction [17]. Thus, we anticipated a positive association between PWSFs and satisfaction with food-related life through the first hypothesis for actor effects (Figure 1):

**H1.**  *PWSFs positively relates to each parent’s satisfaction with food-related life.*

Childcare access, flexible work schedules, and supportive leave policies are a few of the initiatives stemming from PWSFs [23], which can produce positive results for employees’ families [24]. In this line, Schnettler et al. [21] found that the resources parents acquired from the workplace were positively and indirectly related to their adolescent children’s satisfaction with food-related life. However, no partner effects were found between fathers and mothers. Nevertheless, during the pandemic, family members were compelled to stay home due to lockdown measures [2,3], and others chose to stay home due to fear of contagion. This situation led all family members to spend more time together, which may improve the likelihood of crossover effects [17]. On this basis, we propose that one parent’s PWSP may influence not only their children but the other parent’s satisfaction with food-related life, leading to the second hypothesis for partner effects (Figure 1):

**H2.**  *One parent’s PWSFs is linked to (a) the other parent’s and (b) the teenagers’ satisfaction with food-related life.*

The COR theory underscores the importance of social support in meeting needs and safeguarding one’s resources [25], which is particularly relevant in the context of family support. Leung, Mukherjee, and Thurik [26] argue that family support contributes to employees’ positive cycle of accomplishment. This implies that family support can bolster personal resources, leading to enhanced performance in home responsibilities, which may improve individual satisfaction in various life domains [17]. Related to the above, a recent study with a sample of dual-earner families showed that family support was directly and positively related to satisfaction with food-related life in fathers. By contrast, family support was indirectly and positively associated with satisfaction with food-related life in mothers [18]. Therefore, the second hypothesis was posited for actor effects (Figure 1):

**H3.**  *Family support positively relates to each parent’s satisfaction with food-related life.*

According to the COR theory, a contextual resource such as family support may generate personal resources, such as skills (e.g., cooking healthy) that may lead to satisfaction in a close person who shares the same environment [17]. The evidence involving family support and satisfaction with food-related life is scant about the transfer of resources between dyad members [27]. However, before the pandemic, Schnettler et al. [18] found that fathers’ family support crossed over indirectly to the mothers’ satisfaction with food-related life; no partner effects were assessed between parents’ family support and their children’s satisfaction with food-related life. However, evidence shows that adolescents who receive support from their families report higher satisfaction with their food-related life [28]. Thus, we expect partner effects between parents and from parents to the adolescents and posited the following partner effects hypothesis (Figure 1):

**H4.**  *One parent’s family support is positively associated with (a) the other parent’s and (b) the teenagers’ satisfaction with food-related life.*

### 2.2. Perceived Workplace Support for Families, Family Support, and Diet Quality

Following the COR theory, PWSFs may generate personal resources that allow better performance in other domains, such as in the food domain, developing skills, or taking advantage of a flexible schedule to prepare healthy meals [17]. However, the evidence about the association between workplace support and diet quality is also scarce. A recent study by Ererdi et al. [29] with samples of workers from Chile and Spain observed that providing workplace support is advantageous for working parents as it enables them to balance their home obligations effectively. This includes managing care responsibilities and various domestic tasks, particularly food related. In Chile, during the initial year of the pandemic, Schnettler et al. [15] found that the support fathers feel PWSFs was found to have a positive and direct correlation with their dietary habits in households where both parents are employed. Therefore, we posited the following hypothesis for actor effects (Figure 1):

**H5.**  *PWSFs positively relates to each parent’s diet quality.*

Furthermore, it has been reported that employees who receive solid organizational support may allocate resources to benefit their families [30]. Some research suggests that employees can transfer their workplace resources to their partners, leading to improvements in the partner’s resources and more significant commitment to their role as parents [12,31]. Recent evidence shows that PWSFs can also cross over between parents and their children. Schnettler et al. [15] found that fathers’ PWSFs was positively related to the mothers’ and their teenage children’s diet quality during the first year of the pandemic in Chile. Therefore, we posited the following hypothesis for partner effects (Figure 1):

**H6.**  *One parent’s PWSFs is positively linked to (a) the other parent’s and (b) the teenagers’ diet quality.*

Family support remains the most significant form of social support and is crucial for an individual’s physical and mental well-being [32]. Based on the COR theory, family support enables individuals to gain and allocate resources to other areas of their lives [17], such as the food domain. In this regard, research has associated family support with maintaining a healthy lifestyle, for instance, engaging in regular physical activity and following a well-rounded diet [33]. The abundant literature shows a positive association between family/social support and diet quality. For instance, studies with samples of adolescents in the United States and Chile [28,34]; adults in the United States, Spain, France, Brazil, and Chile [6,33,35,36,37]; and older adults from Mexico and the United Kingdom [38,39]. Thus, the following hypothesis was posited for actor effects (Figure 1):

**H7.**  *Family support is positively associated with each parent’s diet quality (actor effects).*

As far as the authors know, there has yet to be any study that has examined the impact of one parent’s family support on the other parent’s and their teenage children’s dietary habits. However, the COR theory posits that crossover can transmit experiences and resources between family members [17]. In this regard, the pandemic has brought about positive food patterns in developed and developing countries due to increased time spent at home and improved familiar interaction [37,40,41,42]. During the pandemic, one beneficial result was that families gathered more often for meals, leading to improved eating habits (e.g., [15,43,44]) and the opportunity for parents and their children to provide emotional support to each other [45,46]. Therefore, it is likely that during the pandemic, the support from one parent’s family impacts the diet quality of the other parent and influences the diet quality of their teenage children. On this basis, the following hypothesis was posed for partner effects (Figure 1):

**H8.**  *One parent’s family support is positively linked to (a) the other parent’s and (b) the teenagers’ diet quality (partner effects).*

### 2.3. Diet Quality and Satisfaction with Food-Related Life

Following the COR theory, a healthy diet can be understood as a resource to achieve high satisfaction with food-related life [7,17]. In this regard, it is well known that in individual-level research, high diet quality has been positively related to greater levels of satisfaction with food-related life in adults (e.g., [7,8,9,47]) and adolescents (e.g., [47,48]). Therefore, the following hypothesis for actor effects was posited (Figure 1):

**H9.**  *Diet quality is positively linked to satisfaction with food-related life for (a) fathers, (b) mothers, and (c) adolescents.*

Recent studies also show crossover or partner effects between these variables, which aligns with the COR theory posited [17]. Studies have shown that mothers’ diet quality crosses over to the fathers, improving the latter satisfaction with food-related life [29], whereas adolescents’ diet quality crosses over to the mothers’ satisfaction with food-related life [18,49]. Therefore, we posited the following hypotheses for partner effects (Figure 1):

**H10.**  *One parent’s diet quality positively relates to (a) the other parent’s and (b) the teenagers’ satisfaction with food-related life.*

**H11.**  *Adolescents’ diet quality positively relates to their parents’ satisfaction with food-related life.*

### 2.4. The Mediating Role of Diet Quality

We also propose that diet quality has a mediating role in the relationship between PWSFs and satisfaction with food-related life and between family support and satisfaction with food-related life.

At an individual level, Schnettler et al. [21] found that mothers’ and fathers’ perceptions of the atmosphere of family meals mediate between resources acquired in the workplace and satisfaction with food-related life in dual-earner families. Regarding interindividual mediating variables, Schnettler et al. [15] found that fathers’ resources acquired in the workplace mediate between their PWSFs and their adolescent children’s diet quality. Schnettler et al. [21] found that adolescents’ perception of the atmosphere of family meals mediate between their parents’ resources acquired in the workplace and the adolescents’ satisfaction with food-related life. Based on this empirical evidence, we argue that similar results may be expected for PWSFs, diet quality, and satisfaction with food-related life. Thus, we posited the following hypothesis that considers actor and partner effects:

**H12.**  *Diet quality mediates between parents’ PWSFs and satisfaction with food-related life for the three members of the family.*

Lastly, at an individual level, Schnettler et al. [49] found that parents’ diet quality mediates between their modeling of healthy eating and satisfaction with food-related life. Schnettler et al. [18] reported that parents’ work-life balance mediates between their family support and satisfaction with food-related life. Although no evidence shows interindividual mediating variables between family support and satisfaction with food-related life, Schnettler et al. [49] found that mothers’ diet quality mediates between their modeling of healthy foods and the fathers’ satisfaction with food-related life. Considering that parents’ modeling of healthy eating can be understood as family support, we argue that similar results may be expected for family support, diet quality, and satisfaction with food-related life. Thus, we posited the last hypothesis that also considers actor and partner effects:

**H13.**  *Diet quality mediates between parents’ family support and satisfaction with food-related life for the three members of the family.*

We are also examining, without making any assumptions, whether the above interactions demonstrate different patterns in fathers and mothers. This is important for several reasons. Firstly, gender norms in Latin American countries are still quite traditional, with women mainly taking on family responsibilities and men being the primary breadwinners [2]. Secondly, women in Chile have taken on the primary responsibility for family meals and, during the pandemic, spend more hours cooking than men [15]. Thirdly, there have been indications of unequal allocation of resources within dual-earner couples, as well as from mothers and fathers to their children [15].

## 3. Materials and Methods

### 3.1. Sample and Procedure

This study is a component of a larger research initiative focusing on the connections between work, family, and food-related aspects of life within Chilean households. The sample size for each city was calculated based on 10 participants for each scale item employed in this research project. This approach is informed by statistical simulation research conducted by Gagne and Hancock [50], who recommended 7–12 participants per item, and by Kyriazos [51], who advocated using 10 participants per item.

To examine the actor and partner effects between parents’ PWSFs and family support, and mothers’, fathers’, and one adolescent child’s diet quality and satisfaction with food-related, a sample is needed to evaluate the degree to which family members influence one another [52]. Hence, the sample inclusion criteria involved families consisting of a mother and father who were either married or cohabiting, employed, and with one adolescent child between the ages of 10 and 16. The sample excluded families in which the parents did not reside together.

The participants were intentionally chosen using quota sampling [53]. Educational institutions were contacted to select samples that accomplish the inclusion criteria and mirror the demographic distribution of families based on the socioeconomic status of each city, as indicated by the CASEN 2015 survey [54], using the Vulnerability Reference Index by Establishment. Upon receiving authorization from educational institutions, families with children aged 10 to 16 were invited to participate in this study via an email dispatched by the academic authorities containing the inclusion criteria in the sample. The response rate was 64% in Temuco and 55% in Santiago. All families who agreed to participate met the inclusion criteria in the sample, so discarding families was unnecessary. Consequently, a non-probabilistic sample was recruited, comprising 860 dual-earner families in Santiago and Temuco (430 families from each city), Chile.

Families who consented to participate were contacted by trained interviewers who had been instructed to provide parents with comprehensive details about the study’s objectives, the format of the questionnaire, and the confidential and anonymous handling of their responses. Families willing to participate in the survey received links to three separate surveys, one for each member of the family. Subsequently, interviewers communicated with the families to address study-related queries and ensure the questionnaires were completed. Previous studies have used this procedure [2,11,15,18,21,49]. Data collection occurred in Santiago between 1 March and 30 July 2021, and between 1 August and 30 December 2021, in Temuco. The online survey for parents included a consent form on the first page, while the survey for adolescents included an informed assent form. All family members were required to indicate their willingness to participate in this study by checking a box. The surveys were stored in separate databases for each family member on the QuestionPro platform (QuestionPro Inc., Mérida, Mexico). After completing the three questionnaires, the family received a 15 USD bank transfer as a token of gratitude for their participation.

A pilot test involving fifty families was conducted in a different city in Chile. The recruitment methods and data-gathering procedures were the same as those used previously, and no adjustments were needed. This research is part of a broader project that explores the relationships between work, family, and food-related aspects within Chilean households. This study received approval from the Ethics Committee at the Universidad de La Frontera (protocol 007/19).

During the COVID-19 pandemic in Chile, various health measures such as quarantines, border closures, social distancing, and mask mandates were implemented. The government implemented a 5-phase strategy consisting of quarantine, transition, preparation, initial opening, and advanced opening, tailored to the health conditions in each commune. This strategy restricted individual activities, mobility, and social interactions [55]. In January 2021, the national vaccination campaign against COVID-19 commenced, gradually easing mandatory confinement in various cities. By June 2021, 80% of adults had received their first vaccine dose. Notably, the Metropolitan Region was under a mandatory lockdown during an important part of the data collection (March to June 2021), while Temuco remained unlocked during the data collection period in this city. Even in locked-down communes, organizations could issue work permits to their employees, resulting in many individuals returning to in-person work [56].

### 3.2. Measures

Parents answered the following measures:

Perceived Workplace Support for Families (PWSFs, [12]): Comprising three items, PWSFs evaluates the extent of workplace support available to an individual for managing their parental responsibilities (e.g., “Overall, at my workplace, there is much understanding of my family demands”). Participants answered each item using a 4-point Likert-type scale (1: never or rarely; 4: always). The research utilized the validated Spanish version, specifically with Chilean employees [15].

Multidimensional Scale of Perceived Social Support (MSPSS, [57]): The MSPSS assesses how individuals perceive the support they receive from their family, friends, and significant other. This study specifically focused on using the four-item Perceived Family Support subscale (e.g., “I can talk about my problems with my family”). Participants were requested to answer each item using a 5-point Likert scale (1: completely disagree; 5: completely agree). The Perceived Family Support subscale in Spanish was utilized to measure Perceived Family Support [58].

The following measures received responses from three family members:

Adapted Healthy Eating Index (AHEI). As a measure of diet quality, this adaptation of the US-HEI [59] was translated into Spanish by Norte and Ortiz [60]. Participants must report how often they consume nine food groups. Norte and Ortiz [60] suggested converting the frequency of consumption for each food group into a score ranging from 0 to 10 based on adherence to daily and weekly food recommendations. Participants report their consumption frequency for the first nine variables and receive a score from 0 to 10 based on specific criteria [60]. The final variable, which pertains to diversity in diet, is determined by assessing how often the nine foods are consumed. Respondents gain two points for meeting the daily recommendations and one point for each weekly recommendation. The scores from the ten variables are then summed to calculate the overall AHEI score, with a maximum score of 100 points. A score above 80 points signifies a “healthy” diet, while scores between 51 and 80 points suggest that a diet “requires changes”. Scores below 50 points indicate “unhealthy” diets [59].

Satisfaction with food-related life scale (SWFoL, [10]). The scale consists of five items and measures an individual’s overall evaluation of their food and eating behaviors in one dimension (e.g., “Food and meals are very positive elements in your life”). Participants answered each item using a 6-point Likert scale (1: completely disagree; 6: completely agree). The research utilized the validated Spanish adaptation of the SWFoL [61], showing good internal consistency in samples of adults, dual-earner couples, and adolescents in Chile [2,18,21,28,47].

The ages of the three family members were recorded, and the adolescents were also inquired about their gender. Parents were asked about their employment status, weekly working hours, and whether they worked remotely or on-site. Additionally, mothers provided the number of family members and children. Based on the work of Schnettler et al. [15], mothers were also asked about the regularity of family meals together as a family (breakfast, lunch, supper, and dinner) and the consumption of ready-to-eat food, ordered food, restaurant/fast food consumption, and homemade meals. Furthermore, mothers were asked about the daily and weekend hours spent cooking by both themselves and their male partners. Lastly, the family’s socioeconomic status (SES) was established by considering the household’s income and size [62].

### 3.3. Data Analysis

This study used SPSS v.23 for descriptive analyses, which included frequency analysis to rule out missing values, outliers, and possible errors in merging the three databases. Bivariate correlation between the main variables was also conducted, as well as t-student and analysis of variance to compare the variables’ scores among family members.

By the methodology proposed by Claxton, DeLuca, and van Dulmen [63], we conducted a dyadic confirmatory factor analysis (CFA) to evaluate the underlying structure and psychometric characteristics of each scale employed in this study. A triadic approach was followed in the case of the satisfaction with food-related life scale. The internal consistency was assessed using the Omega coefficient [64]. Convergent validity was established by scrutinizing the standardized factor loadings of each scale (ideally > 0.5), their statistical significance, and the average variance extracted (AVE, values > 0.5). Discriminant validity was verified by comparing the AVE for each scale with the square of the correlation between the factor scores of the scales [65].

The Actor–Partner Interdependence Model (APIM) was tested with distinguishable dyads through covariance-based structural equation modeling (SEM), as described by Kenny, Kashy, and Cook [20]. The analysis included examining actor effects, which demonstrate the relationships between variables for a specific family member, and partner effects, which demonstrate the relationships between variables from one family member to another. Parents and adolescents were considered actors and partners. The study examined the actor and partner effects of mothers’ and fathers’ PWSFs, Perceived Family Support (PFS), and the three family members’ diet quality and satisfaction with food-related life (SWFoL). The mediation Actor–Partner Interdependence Model also analyzed the potential mediating role of each family member’s diet.

The APIM manages the interdependence among family members. Specifically, the correlation between each parent’s PWSFs and PFS was utilized to regulate the impact of PWSFs and PFS between the two parents. To account for other factors that could affect the relationship between partners, the residual errors of the dependent variables (AHEI and SWFoL) for the three family members were correlated, following the approach outlined by Kenny, Kashy, and Cook [20]. Furthermore, this study included controls for various variables, including the ages of the family members, both parents’ employment type and weekly working hours, family socioeconomic status (SES), number of children, and city of residence. The controlled variables directly affected the dependent variables of the three family members (AHEI and SWFoL).

The CFA and SEM were carried out using Mplus 8.8. The CFA and SEM were conducted using Mplus 8.8. The parameters were estimated using weighted least square mean and variance adjusted (WLSMV) to account for the ordinal scale of the items by utilizing the polychoric correlation matrix. Evaluation of the CFA and SEM model fit relied on the Tucker–Lewis index (TLI) and the comparative fit index (CFI), where values exceeding 0.95 denoted a favorable fit and those surpassing 0.90 indicated an acceptable fit. Furthermore, the root mean square error of approximation (RMSEA) was employed to appraise the fit, with values below 0.06 signifying a good fit and those below 0.08 indicating an acceptable fit [66]. The evaluation of the mediating role of diet quality was conducted through SEM employing a bias-corrected (BC) bootstrap confidence interval with 1000 samples, following the framework established by [67].

## 4. Results

### 4.1. Sample Description

The data presented in Table 1 outline the characteristics of the sample. The sample consists of 860 families, divided evenly between Temuco and Santiago, with the majority displaying a middle socioeconomic status. On average, these families consist of four individuals, encompassing two children. The preponderance of fathers and mothers were gainfully employed, with over 70% of fathers and slightly over 50% of mothers maintaining full-time positions. Additionally, the predominant number of fathers and nearly 60% of mothers were engaged in occupations requiring direct interpersonal interaction. Family members typically shared four meals at least twice weekly, mostly at supper time, and consumed homemade food regularly. Mothers invested more time cooking than fathers during the week and on weekends.

### 4.2. Scores and Correlations between the Main Variables

Table 2 displays the mean scores and relationships for the PWSFs and PFS of parents, as well as those of the three family members’ diet quality (AHEI) and SWFoL. Most associations were significant and aligned with expectations other than those among mothers’ PWFS and their and the adolescents’ AHEI. Also, between fathers’ PWFS and the mothers’ and adolescents’ AHEI, fathers’ PWFS and the adolescents’ SWFoL, and fathers’ PFS and mothers’ AHEI. Mothers achieved notably higher scores than fathers in PWSFs (t = 3.7, *p* < 0.001), whereas fathers achieved significantly higher scores than mothers in PFS (t = −4.6, *p* < 0.001). Fathers scored significantly lower than adolescents and mothers in AHEI (F = 31.7, *p* < 0.001), whereas mothers did not differ from adolescents. According to Kennedy et al. [62] proposed cut-off point, the average AHEI scores of the three family members indicated that their diet “requires changes”. Adolescents had significantly higher SWFoL scores than their parents (F = 31.4, *p* < 0.001), and fathers had significantly higher scores than mothers.

### 4.3. APIM Results

#### 4.3.1. Confirmatory Factor Analysis and Psychometric Properties

The dyadic CFAs showed that the Perceived Workplace Support for Families scale (RMSEA = 0.04; CFI = 0.99; TLI = 0.99) and the Perceived Family Support subscale (RMSEA = 0.09; CFI = 0.99; TLI = 0.99) demonstrated good or acceptable fit to the data for parents. Similarly, the satisfaction with food-related life scale (RMSEA = 0.09; CFI = 0.99; TLI = 0.98) showed acceptable results for mothers, fathers, and adolescents. All the scales exhibited strong reliability, with Omega coefficients ranging from 0.87 to 0.94 and AVE values surpassing 0.50. Convergent validity was supported by the substantial size of the factor loadings, which were all statistically significant and exceeded 0.50. Furthermore, the AVE values exceeded the square correlation between the factorial scores of the scales, demonstrating discriminant validity (Table 3).

#### 4.3.2. APIM Goodness of Fit and Control Variables

**Figure 2:**
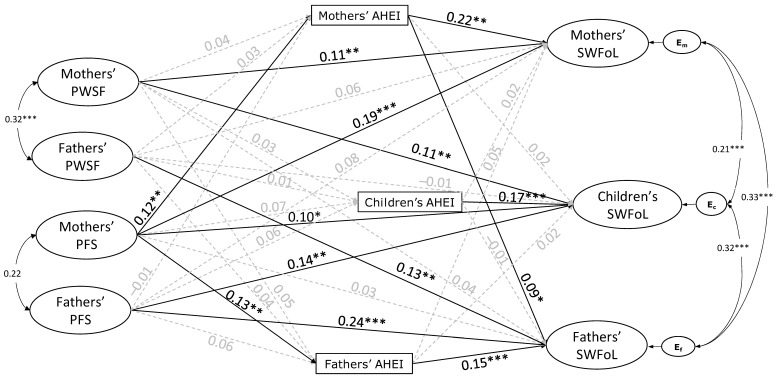
Actor–Partner Interdependence Model of the effect of Perceived Workplace Support for Families (PWSFs), Perceived Family Support (PFS), diet quality (AHEI), and satisfaction with food-related life (SWFoL) in dual-earner parents with teenagers. The numbers over or below the arrows are the standardized path coefficients obtained through the mediation APIM. E_m_, E_c_, and E_f_: residual errors on SWFaL for mothers, teenagers, and fathers, respectively. * *p* < 0.05 ** *p* < 0.01 *** *p* < 0.001. The path diagram did not show the control for the effects of the three family members’ age, both parents’ number of working hours and type of employment, the family SES, the number of children, and the city of residence on the dependent variables of the three family members (AHEI and SWFoL).

The results from the structural model estimation are shown in Figure 2. For the Perceived Workplace Support for Families scale, all factor loadings were significant (*p* < 0.001) and over 0.83 for mothers and over 0.87 for fathers. The AVE values were good (mothers = 0.76, fathers = 0.79). For the Perceived Family Support subscale, also all factor loadings were significant (*p* < 0.001) and over 0.86 for mothers and over 0.89 for fathers, and the AVE values were good (mothers = 0.80, fathers = 0.85). For the Satisfaction with food-related Life scale, the factor loadings were significant (*p* < 0.001) and above 0.62 for mothers, above 0.71 for fathers, and above 0.67 for adolescents (*p* < 0.001). The AVE values were also good (mothers = 0.59, fathers = 0.67, adolescents = 0.63).

The model that evaluated the APIM association between mother’s and father’s PWFS, Perceived Family Support, and the three family members’ diet quality (measured by the AHEI) and SWFoL had a good fit with the data (CFI = 0.98; TLI = 0.98; RMSEA = 0.04). A significant association (covariance) was discovered between the PWSFs (r = 0.32, *p* < 0.001) of mothers and fathers. The association between parents’ PFS (r = 0.22, *p* = 0.08) was not statistically significant. However, there were significant correlations between the residual errors of mothers’ and fathers’ SWFoL (r = 0.33, *p* < 0.001), between mothers’ and adolescents’ SWFoL (r = 0.21, *p* < 0.001), and between fathers’ and adolescents’ SWFoL (r = 0.32, *p* < 0.001). Additionally, significant correlations were found between the residual errors of mothers’ and fathers’ (r = 0.44, *p* < 0.001), between mothers’ and adolescents’ (r = 0.52, *p* < 0.001), and between fathers’ and adolescents’ (r = 0.40, *p* < 0.001) diet quality (AHEI).

Most of the control variables did not significantly affect the model. The family SES (γ = 0.08, *p* = 0.03) and the mothers’ age (γ = 0.13, *p* = 0.02) positively affected their SWFoL. The adolescents’ age negatively affected their (γ = −0.10, *p* = 0.004), the fathers’ (γ = −0.07, *p* = 0.03), and the mothers’ (γ = −0.08, *p* = 0.03) SWFoL.

#### 4.3.3. Testing Actor–Partner Hypotheses

##### Perceived Workplace Support for Families, Family Support, and Satisfaction with Food-Related Life

As depicted in Figure 2, the standardized path coefficients show that the fathers’ (γ = 0.13 *p* = 0.001) and the mothers’ (γ = 0.11, *p* = 0.002) PWSFs were significantly associated with their SWFoL. These findings supported H1.

Neither the fathers’ PWSFs was significantly associated with the mothers’ SWFoL (γ = 0.06, *p* = 0.10), nor the mothers’ PWSFs was significantly associated with the fathers’ SWFoL (γ = 0.04, *p* = 0.23). While fathers’ PWSFs was not related to the adolescents’ SWFoL (γ = −0.01, *p* = 0.88), mothers’ PWSFs was positively associated with the adolescents’ SWFoL (γ = 0.11, *p* = 0.005). Therefore, H2a was not supported, and H2b was supported only for mothers.

Fathers’ (γ = 0.24, *p* < 0.001) and mothers’ (γ = 0.19, *p* < 0.001) PFS were positively associated with their SWFoL; thus, H3 was supported.

Neither the fathers’ PFS was associated with the mothers’ SWFoL (γ = 0.08, *p* = 0.07), nor the mothers’ PFS was associated with the fathers’ SWFoL (γ = 0.03, *p* = 0.48). Fathers’ (γ = 0.14, *p* = 0.002) and mothers’ (γ = 0.10, *p* = 0.02) PFS were positively associated with the adolescents’ SWFoL. These findings did not support H4a, while they supported H4b for fathers and mothers.

##### Perceived Workplace Support for Families, Family Support, and Diet Quality

Neither fathers’ (γ = 0.04, *p* = 0.33) nor mothers’ (γ = 0.04, *p* = 0.34) PWSFs were significantly associated with their diet quality. These findings did not support H5.

Neither the fathers’ PWSFs was significantly associated with the mother’s diet quality (γ = 0.03, *p* = 0.34) nor the mothers’ PWSFs was significantly associated with the father’s diet quality (γ = 0.05, *p* = 0.24). Likewise, neither the fathers’ (γ = 0.01, *p* = 0.87) nor the mother’s (γ = 0.03, *p* = 0.52) PWSFs were associated with adolescents’ diet quality. These findings did not support H6.

While fathers’ PFS was not significantly associated with their diet quality (γ = 0.06, *p* = 0.14), mothers’ PFS (γ = 0.12, *p* = 0.003) was positively related to their diet quality, supporting H7 only for mothers.

Fathers’ PFS was not significantly associated with the mother’s diet quality (γ = −0.01, *p* = 0.75); by contrast, mothers’ PFS was positively associated with the fathers’ diet quality (γ = 0.13, *p* = 0.002). Neither fathers’ (γ = 0.06, *p* = 0.16) nor mothers’ (γ = 0.07, *p* = 0.08) PFS were significantly associated with the adolescents’ diet quality. These findings supported H8a only for mothers, while they did not support H8b.

##### Diet Quality and Satisfaction with Food-Related Life

Fathers’ (γ = 0.15, *p* < 0.001), mothers’ (γ = 0.22, *p* < 0.001), and adolescents’ (γ = 0.17, *p* < 0.001) diet quality were positively related to their SWFoL, thus supporting H9.

Fathers’ diet quality was not statistically associated with the mothers’ SWFoL (γ = 0.05, *p* = 0.20). By contrast, the mothers’ diet quality was positively associated with the fathers’ SWFoL (γ = 0.09, *p* = 0.04). Neither fathers’ (γ = 0.02, *p* = 0.64) nor mothers’ (γ = 0.02, *p* = 0.69) diet quality was significantly related to the adolescents’ SWFoL. These findings supported H10a only for mothers, while they did not support H10b.

However, adolescents’ diet quality was not related to their fathers’ (γ = −0.01, *p* = 0.78) and mothers’ (γ = 0.02, *p* = 0.58) SWFoL; thus, H11 was not supported.

In summary, the hypotheses testing actor effects supported were H1, H3, H5, and H9, whereas Hypothesis H7 was partially supported. By contrast, most hypotheses testing partner effects were partially supported (H2, H4, H8, and H10) or were not supported (H6 and H11).

### 4.4. Testing the Mediating Role of the Diet Quality

In this research, the mediating role of the diet quality of three family members was examined about the influence of parents’ PWSFs (H12) and PFS (H13) on the three family members’ SWFoL. The mediating role of diet quality was only tested for the significant relationships previously found. This study found that mothers’ diet quality plays a role in mediating the link between their PFS and SWFoL (standardized indirect effect = 0.02, *p* = 0.007). The same mediation effect was observed for fathers’ diet quality in the relationship between mothers’ PFS and the fathers’ SWFoL (standardized indirect effect = 0.02, *p* = 0.01). No other significant mediating roles of diet quality were identified. These results did not align with hypothesis H11 but partially supported hypothesis H12.

In summary, diet quality did not show a mediating role between the parents’ PWSFs and the three family members’ SWFoL. Between parents’ PFS and the three family members’ SWFoL, mothers’ diet quality showed one intraindividual mediating role (between mothers’ PFS and SWFoL). In contrast, fathers’ diet quality showed one interindividual mediating role (between mothers’ PFS and fathers’ SWFoL).

## 5. Discussion

This study focused on revisiting the role of parents’ PWSFs and family support on the diet quality and satisfaction with food-related life for parents and one teenage child in the second year of the pandemic. Using the mediation APIM, we found that mothers’ and fathers’ workplace support for families was directly and positively associated with their satisfaction with food-related life. Only mothers’ workplace support for families crossed over to their adolescent children, improving their satisfaction with food-related life. Neither parents’ workplace support for families influences their and their teenage children’s diet quality. Parents’ family support was positively and directly associated with their satisfaction with food-related life and the teenagers’. Only the support of the mothers’ families improved their diet quality while enhancing the fathers’ diet quality, but not vice versa. The three family members’ diet quality was positively linked to their satisfaction with food-related life. However, only the mothers’ diet quality crossed over to the fathers, improving their satisfaction with food-related life. Lastly, intraindividual and interindividual mediating roles of parents’ diet quality were found.

### 5.1. Perceived Workplace Support for Families, Family Support, and Satisfaction with Food-Related Life

According to the COR theory [30], PWSFs includes resources to assist employees in managing the demands of their family life (e.g., food-related tasks) more effectually [22,24,29,31], this might, in turn, increase the person’s overall satisfaction with food-related life [24]. Hence, we anticipated that the PWSFs of each parent would positively correlate with their satisfaction with food-related life (H1). This hypothesis was supported for mothers and fathers (actor effects), which is a novel finding that fills one of the research gaps identified in this study, that is, policies promoting a family-friendly work environment (i.e., PWSFs [22]) enhance employees’ satisfaction with food-related life. Our findings align with a pre-pandemic study in Chile that found resources parents acquire from the workplace are positively related to their satisfaction with food-related life because these types of resources may cultivate individual assets to be utilized in the realm of food, improving the performance in this domain which in turn enhance satisfaction with food-related life [17,21]. However, the study above did not distinguish the type of workplace resource that improves satisfaction with food-related life. Thus, our findings further build upon this understanding, identifying a specific kind of workplace resource that can improve satisfaction with food-related life, irrespective of the parent’s gender.

We also expected that one parent’s PWSFs would be positively linked to the other parent’s (H2a) and the teenager’s (H2b) satisfaction with food-related life (partner effects). However, only H2b was partially supported. Namely, no partner effects were discovered between parents, while only mothers’ PWSFs crossed over to the adolescents’ satisfaction with food-related life. Although the lack of crossover effects between parents aligns with a previous study [21], we posed this hypothesis on the basis that due to the pandemic, family members were required to remain at home due to the implementation of lockdown measures [2,3]. However, unlike the pandemic’s first year, most fathers worked face-to-face in the sample under study during the second year. Therefore, the likelihood that one parent’s PWSFs crossed over to the other parent’s satisfaction with food-related life is low. In part, this result may also be related to the fact that the frequency of family meals was similar to pre-pandemic studies in Chile (e.g., [47]), which confirms that the crossover process to satisfaction with food-related life between parents does not occur when they have a low frequency of shared meals (e.g., [21]). The direct partner effect from mothers’ PWSFs to the adolescents’ satisfaction with food-related life is a novel result that contributes to the knowledge because previous studies [15,19] reported that resources that parents acquired from the work domain indirectly relate to their children’s well-being. Although more research is needed to explain this direct partner effect better, a possible explanation may be associated with the significantly higher PWSFs that mothers reported compared to fathers. Therefore, mothers may have more resources to invest in activities that improve the adolescents’ satisfaction with food-related life.

As per the COR theory, family resources have the potential to enhance personal resources, including skills, leading to positive outcomes in home role performance and the well-being of both the employee and their family [17]. We hypothesized that each parent’s family support would positively affect satisfaction with food-related life (H3, actor effects). This hypothesis was supported for mothers and fathers. In the case of fathers, this finding aligns with a study conducted before the pandemic [18]. By contrast, in the above study, family support was only indirectly associated with satisfaction with food-related life in working mothers through work-life balance. Therefore, in the case of mothers, our results expand on the knowledge, showing that the association between family support and satisfaction with food-related life may also be direct in female employees. It is likely that under the pandemic’s conditions, family support perceived by mothers generates feelings of balance, which helps that family support directly contributes to an enhancement of mothers’ satisfaction with food-related life. Thus, if one parent perceives emotional support from the other parent and their adolescent children, they will access resources directed toward the food domain, enhancing their satisfaction with food-related life.

We also proposed that one parent’s family support would be positively related to the other parent’ (H4a) and the teenagers’ (H4b) satisfaction with food-related life (partner effects). Hypothesis 4a was not supported; namely, neither parent’s family support crossed over to the other parent’s satisfaction with food-related life. This finding confirms pre-pandemic evidence showing no direct crossover effects between members of a couple’s family support and satisfaction with food-related life [18]. One feasible explanation for the lack of crossover effects may be related to the fact that during the second year of the pandemic, in many households, parents did not have much time to spend together during the day. By contrast, Hypothesis (H4b) was supported for mothers and fathers; parents’ family support crossed over to the adolescents, improving their satisfaction with food-related life. Thus, this is the first study that shows that family support perceived by parents can be passed on to their adolescent children. Whereas these findings also fill other of the gaps in the literature detected in the present study, they also underscore the relevance of family support for satisfaction with food-related life in working parents with adolescents in the second year of the global health crisis.

### 5.2. Perceived Workplace Support for Families, Family Support, and Diet Quality

Contrary to the expectations [12,15,31], mothers’ and fathers’ PWSFs were not significantly linked to their diet quality (H5 not supported, actor effects). These findings are inconsistent with those reported by Schnettler et al. [15] during the first year of the pandemic, who found that fathers’ and mothers’ PWSFs was directly and indirectly positively associated with their diet quality. In the case of fathers, a possible explanation may be their lower presence at home during the second year of the pandemic compared to the first year, which prevented them from being involved in food-related tasks that may have improved their diet quality. Although mothers reported a higher level of PWSFs than fathers, it may be possible that it was not enough to allow mothers to take care of their own diet quality. A high proportion of mothers worked from home in our sample, meaning they had to cope with their work demands and all family demands without the fathers’ help. Thus, mothers likely used PWSFs in multiple work and family tasks rather than use this resource to improve their diet quality.

Similarly, one parent’s PWSFs did not cross over to the other parent’s and their adolescent children’s diet quality (H6 not supported, partner effects). These findings also contradict results reported by Schnettler et al. [15] in the first year of the pandemic regarding the relevance of fathers’ PWSFs to improve their, the mothers’, and their adolescent children’s diet quality. Therefore, fathers were less likely to use resources from the workplace to enhance their family members’ diet quality when they returned to working on-site in the second year of the pandemic. Regardless of the above, the lack of partner effects from parents’ WPSF to each other and the adolescents’ diet quality may be explained on the basis of the non-significant actor effects between parents’ PWSFs and diet quality. When actor effects exhibit low magnitudes or lack statistical significance, it is indicative that partner effects are unlikely to be present [68]. Hence, taken together, our findings show that resources parents acquired from the workplace were not invested in the food domain to improve their and the adolescents’ diet quality.

We also hypothesized that each parent’s family support would positively affect diet quality (H7, actor effects). This hypothesis was supported only for mothers, consistent with prior research with samples of different ages and countries [6,33,34,35,36,37,38,39]. Considering that in Chile, traditional gender roles still prevail, and mothers are the primary individuals in charge of tasks related to food [47], this finding is consistent with Leung, Mukherjee, and Thurik [25] suggestion that family support creates a feeling of role accomplishment, that in parallel allow mothers to improve their diet quality. Thus, the father’s role as the leading provider for the household may explain the null association between the father’s family support and their diet quality. These findings also align with those reported by Lima Tribst, Tramontt, and Baraldi [37] in Brazil, which revealed that individuals who improved their dietary habits during the pandemic tended to cook more frequently, as was the case for mothers in our sample (see Table 1).

We also expected partner effects., i.e., one parent’s family support would be positively related to the other parent’s (H8a) and the teenagers’ (H8b) diet quality. However, H8a was supported only for mothers, and H8b was not supported. Namely, mothers’ family support crossed over to the fathers, who improved their diet quality, but not the other way around. In contrast, neither parent’s family support crossed over to the teenagers’ diet quality. It is possible that this could be attributed to how often families shared meals during the second year of the pandemic, which is similar to studies conducted in Chile before the pandemic [47]. Therefore, there was a lower likelihood that all family members share a healthy meal (e.g., [15,43,44]) and provide emotional support to each other [45,46] than during the first year of the pandemic. However, the positive crossover from mothers’ family support to the fathers’ diet quality may be related to the lower score of fathers in the AHEI. Although the scores of the three family members suggested that their diets needed adjustments, mothers and adolescents had comparable scores in the AHEI. Therefore, it is feasible that mothers were more worried about their male partners’ diet quality rather than the adolescents’ diet quality and transferred the resources gained from family support to improve the fathers’ diet quality. Regardless of the above, this result expands knowledge, showing that family support not only enhances the diet quality of those who receive it but can also be used to improve the diet quality of other family members, such as the spouse.

### 5.3. Diet Quality and Satisfaction with Food-Related Life

Based on well-known empirical evidence that related intraindividual healthier eating habits to higher levels of well-being in the food domain, we expected that diet quality would be positively related to satisfaction with food-related life for fathers (H9a), mothers (H9b), and adolescents (H9c). This hypothesis was supported for the three family members, which aligns with earlier research in adult (e.g., [7,8,9,47]) and adolescent samples (e.g., [47,48]).

Based on previous studies [18,49] showing partner effects, we expected that one parent’s diet quality would be positively associated with the other parent’s (H10a) and the teenagers’ (H10b) satisfaction with food-related life. Hypothesis H10a was supported only for mothers; a partner effect from mothers’ diet quality to the fathers’ satisfaction with food-related life was found, but not the other way around. These results confirm those Schnettler et al. [49] reported in the initial year of the pandemic. These authors explained their findings based on parents’ differences in diet quality. Namely, as the fathers’ diet quality was worse than the mothers’, the fathers’ dietary habits did not significantly impact the mothers’ satisfaction with food-related life. In contrast, the opposite trend was observed between mothers’ diet quality and fathers’ satisfaction with food-related life. Therefore, based on our findings and those of Schnettler et al. [49], it is possible to suggest that as long as fathers have a lower diet quality than mothers, it seems unlikely that fathers’ eating habits would be significantly associated with mothers’ satisfaction with food-related life. This is especially true when mothers use resources from family support to improve their partners’ diet quality, as was found in our study. In this case, it could also be viewed as a failure for mothers, as they are not fully fulfilling their role as the primary person responsible for providing food for their family [47]. H10b was not supported for mothers and fathers, which aligns with earlier research demonstrating that adolescents’ satisfaction with food-related life is unrelated to their parents’ diet quality [18,49]. Contrary to the expectations, adolescents’ diet quality did not cross over to their parents’ satisfaction with food-related life (H11 not supported). Whereas in the case of fathers, our findings confirm results reported by Schnettler et al. [49], in the case of mothers, these findings contradict earlier studies [18,49] in which adolescents’ diet quality crossed over to the mothers’ satisfaction with food-related life. Additional research is required to discern the specific conditions under which this partner effect manifests. One potential rationale in the present study could be linked to the maternal high concern about the father’s lower diet quality.

### 5.4. The Mediating Role of Diet Quality

The last two hypotheses examined whether the quality of diet mediates the relationship between each parent’s PWSFs (H12) and family support (H13) and the satisfaction of the three family members with their food-related lives. Contrary to expectations, neither parent’s diet quality mediated between their PWSFs and the three family members’ satisfaction with food-related life (H12 not supported). These findings align with the absence of actor and partner impacts from fathers’ and mothers’ PWSFs on diet quality. Consequently, these results suggest that the resources obtained by parents from PWSFs did not contribute to improved performance in food-related tasks aimed at enhancing the dietary quality of family members and, in this way, improve their family’s satisfaction with food-related life.

We anticipated that diet quality would mediate between parental family support and satisfaction with food-related life for the three family members (H13, actor and partner effects). This hypothesis received partial support, entailing two significant intermediary roles, one at the individual and one at the interpersonal levels. The first shows that support from mothers’ families indirectly and positively impacted their satisfaction with food-related life by enhancing their diet quality. This finding partially aligns with evidence showing that parents’ diet quality mediates between their modeling of healthy eating and satisfaction with food-related life during the first year of the pandemic [49]. The absence of a mediating role of paternal dietary quality in this study suggests that the heightened participation of fathers in food-related responsibilities observed during the initial year of the pandemic (e.g., [44]) reverted to pre-pandemic levels. The second one shows that the support of the mothers’ family indirectly and positively affected the fathers’ satisfaction with food-related life by enhancing their diet quality. This finding aligns with results reported by Schnettler et al. [49] in the initial year of the pandemic. Namely, mothers’ diet quality mediates between their modeling of healthy foods and the fathers’ satisfaction with food-related life. Notably, our findings expand on the knowledge of variables that may be associated indirectly with satisfaction with food-related life via improving diet quality at intraindividual and interindividual levels. In addition, they also fill one of the research gaps in the present study, that is, showing that diet quality may mediate between family support and satisfaction with food-related life.

In summary, both parents’ satisfaction with food-related life was positively influenced by two direct effects (from their PWSFs and family support). In contrast, the diet quality of both mothers and fathers was positively influenced by the support from the mother’s family, which in turn led to an improvement in each parent’s satisfaction with food-related life. Adolescents’ satisfaction with food-related life received the direct interindividual effects of mothers’ PWSFs and parents’ family support. Thus, it is possible to suggest that the direct actor and partner effects from one parent’s PWSFs or family support to her/his, the other parent, and the adolescents’ satisfaction with food-related life may be associated with the social dimension of food [11].

## 6. Limitations

This study presents limitations that warrant attention in future research. Firstly, using a cross-sectional design in this study limits the ability to infer causality between variables. As a result, it is recommended that future research endeavors consider employing longitudinal, experimental, or quasi-experimental designs to provide a more robust foundation for establishing causal relationships. The lack of randomness in the sample limits the ability to apply the findings to dual-earner parents with adolescents in Chile. Furthermore, the sample only included families with adolescents between 10 and 16. This inclusion criterion biased the results and neglected the relationships between the variables in families with older adolescents, who may provide more support to their parents. Therefore, upcoming research should employ a probabilistic sample and assess the impact of the factors included in this study on the well-being of older adolescents. Furthermore, this study’s restriction to two cities in a single developing country in Latin America raises concerns about its representativeness because results may be biased due to Chilean society’s socioeconomic and cultural characteristics, such as income and gender inequity. Consequently, cross-cultural studies that include nations and societies with different levels of gender equality and economic progress are warranted. Furthermore, although the city of residence was controlled for in the APIM, showing no significant effects on the model’s results, data was collected at different time points in Santiago and Temuco cities, potentially influencing the outcomes due to the distinct pandemic conditions experienced in each city during 2021. Another limitation pertains to diet quality measurement, as the AHEI does not encompass all food groups or account for the quantity consumed for each. Lastly, the absence of inquiries regarding working conditions at both the workplace and home in the questionnaire is a further limitation, which may affect the results by having workers in the sample with adequate working conditions and others not, such as differences in computer equipment, having or not having to share this equipment with their children, or having access to good-quality internet. This matter must be considered in future research efforts, particularly when studying samples of employees working from home and on-site.

## 7. Conclusions

Regardless of the limitations, our findings remain pertinent to future public health crises and the experiences of dual-earner parents with adolescent children in the post-pandemic era, notwithstanding the prevailing constraints and the end of the COVID-19 pandemic. It is important to note that this study is the first to simultaneously assess the actor and partner effects of Perceived Workplace Support for Families, family support, diet quality, and satisfaction with food-related life in different family members. The main contributions to the literature are significant, as we have shown that parents’ PWSFs has a direct impact on their and the adolescents’ satisfaction with food-related life, that family support can influence satisfaction with food-related life through diet quality improvement, and that parents’ family support can also positively affect their adolescent children’s satisfaction with food-related life.

Our research also indicates that the impact of workplace support for families on diet quality varied in Chile between the first and second years of the pandemic. A study during the first year highlighted the importance of fathers’ PWSFs in improving their own, mothers’, and adolescents’ diet quality [15]. However, our findings during the second year show that the influence of both parents’ workplace support for families on the family’s diet quality was negligible. This could be due to many families returning to pre-pandemic routines, with mothers taking on the primary responsibility for cooking while continuing to work and most fathers returning to in-person work. Our results indicate that only mothers’ family support was positively correlated to their own and the fathers’ diet quality. While both types of support directly contributed to the satisfaction with food-related life for all family members, it seems that family support was relatively more significant than workplace support for families. Namely, both parents’ family support improved their and their children’s satisfaction with food-related life. In contrast, both parents’ PWSFs enhanced their satisfaction with food-related life, and only mothers’ PWSFs improved the adolescents’ satisfaction with food-related life. Although our findings indicate that both parents can pass on resources to each other and their offspring, they also underscore the importance of mothers as resource providers.

Our findings have implications in theory, research, and practical applications. The theoretical implications highlight the importance of family support in the second year of the health crisis. Family support improved diet quality and satisfaction with food-related life in those who received it and those who provided it (i.e., the partner and adolescents). This suggests a positive cycle among family members. Furthermore, our results indicate that the COR theory-widely used to study the work-family interface (e.g., [26])—is suitable for analyzing the use and transference of contextual resources (i.e., both types of support) to improve the food domain’s nutritional and well-being aspects.

Research implications underscore the need to assess if mothers are the leading resource providers in cultures with more gender equity. Our results indicate that both forms of support directly impacted the satisfaction with the food-related lives of the three family members through the social aspect of food. Additional research is required to distinguish the particular elements of food-related life, such as meal planning, grocery shopping, meal preparation, consumption, and waste management, in which the social dimension of food improves the three family members’ satisfaction with food-related life. It is also crucial to thoroughly evaluate a diverse range of support channels, encompassing both emotional and practical forms, such as the assistance provided by partners in the family sphere and the support extended by supervisors and co-workers within the professional realm. Furthermore, the diets of the three family members needed to be modified to improve their health, although they almost consumed homemade foods daily. Thus, future studies should assess parents’ cooking skills and the type of prepared homemade foods.

From a practical standpoint, our research underscores the importance of promoting family support within individual households and at a societal level. For instance, schools could play a crucial role in teaching children the value of family support from an early age, irrespective of gender. Policymakers, too, have a significant role to play. They can make a tangible difference by encouraging organizations to provide workplace support for families, particularly those with parents. Health and labor authorities should also take note of creating initiatives to improve the dietary habits of workers and their families, with a specific focus on male workers. These practical measures can empower families to make healthier choices and enhance their food-related well-being.

## Figures and Tables

**Table 1:** Sample characteristics.

Characteristic	Total Sample
Age [Mean (SD)]	
Mother	40 (7)
Father	43 (8)
Adolescent	13 (2)
Adolescents’ gender (%)	
Male	50.7
Female	49.3
Number of family members [Mean (SD)]	4 (1)
Number of children [Mean (SD)]	2 (1)
Socioeconomic status (%)	
High	5.0
Middle	81.3
Low	13.7
Mothers’ type of employment (%)	
Employee	67.6
Self-employed	32.4
Fathers’ type of employment (%)	
Employee	74.2
Self-employed	25.8
Mothers’ working hours (%)	
45 h per week	52.2
Less than 45 h per week	47.8
Fathers’ working hours (%)	
45 h per week	73.5
Less than 45 h per week	26.5
Mothers’ place of working (%)	
Remote	40.3
Face-to-face	59.7
Fathers’ place of working (%)	
Remote	18.8
Face-to-face	81.2
Number of days/week couples ate together [Mean (SD)]	
Breakfast	3 (3)
Lunch	5 (2)
Super	5 (3)
Dinner	3 (3)
Number of days families eat different types of foods [Mean (SD)]	
Homemade foods	6 (1)
Buy ready-to-eat food	0.6 (1)
Order food at home	1 (1)
Eat at restaurants	0.3 (1)
Eat at fast-food outlets	0.5 (1)
Number of hours per day spent cooking during the week [Mean (SD)]	
Mother	3 (1)
Father	1 (1)
Number of hours per day spent cooking on the weekend [Mean (SD)]	
Mother	3 (2)
Father	2 (1)

SD: Standard deviation.

**Table 2:** Descriptive statistics and correlations (r) for Perceived Workplace Support for Families (PWSFs), Perceived Family Support (PFS), Diet Quality (AHEI), and satisfaction with food-related life (SWFoL) in different-sex dual-earner parents with teenagers (n = 860).

	M (SD)	Correlations									
		1	2	3	4	5	6	7	8	9	10
1. Mothers’ PWSFs	8.1 (2.7)	1	0.28 ***	0.09 *	0.13 **	0.08 *	0.06	0.04	0.15 ***	0.11 **	0.13 ***
2. Fathers’ PWSFs	7.6 (2.9)		1	0.13 ***	0.18 ***	0.09 *	0.06	0.04	0.14 **	0.18 ***	0.06
3. Mothers’ PFS	16.4 (3.5)			1	0.33 **	0.14 ***	0.11 **	0.09 **	0.23 ***	0.14 ***	0.13 ***
4. Fathers’ PFS	17.2 (3.2)				1	0.11 **	0.05	0.09 **	0.17 ***	0.24 ***	0.12 ***
5. Fathers’ AHEI	59.4 (13.9)					1	0.45 ***	0.41 ***	0.20 ***	0.23 ***	0.12 **
6. Mothers’ AHEI	64.2 (13.3)						1	0.52 ***	0.25 ***	0.17 ***	0.12 ***
7. Adolescents’ AHEI	63.6 (14.0)							1	0.17 ***	0.13 ***	0.20 ***
8. Mothers’ SWFoL	21.6 (4.5)								1	0.35 ***	0.23 ***
9. Fathers’ SWFoL	22.6 (4.7)									1	0.30 ***
10. Adolescents’ SWFoL	23.4 (4.8)										1

* Correlation is significant at the 0.05 level (2-tailed). ** Correlation is significant at the 0.01 level (2-tailed). *** Correlation is significant at the 0.001 level (2-tailed). M: Mean. SD: Standard deviation.

**Table 3:** Results of the dyadic and triadic confirmatory factor analysis for the Perceived Workplace Support for Families scale (PWSFs), Perceived Family Support subscale (PFS), and the satisfaction with food-related life scale (SWFoL).

Scale	Loadings Range (Min–Max)	Omega	AVE	1	2	3	4	5	6	7
1. Mothers’ PWSFs	0.85–0.91	0.90	0.75	-	0.14	0.01	0.02	0.02	0.02	0.02
2. Fathers’ PWSFs	0.86–0.90	0.92	0.78	0.37 ***	-	0.03	0.04	0.02	0.04	0.01
3. Mothers’ PFS	0.87–0.92	0.94	0.80	0.11 **	0.16 ***	-	0.24	0.06	0.04	0.03
4. Fathers’ PFS	0.90–0.94	0.94	0.86	0.14 ***	0.20 ***	0.49 ***	-	0.04	0.07	0.04
5. Mothers’ SWFoL	0.60–0.91	0.87	0.59	0.14 ***	0.15 ***	0.25 ***	0.19 ***	-	0.23	0.12
6. Fathers’ SWFoL	0.69–0.94	0.91	0.66	0.13 ***	0.19 ***	0.19 ***	0.27 ***	0.48 ***	-	0.19
7. Adolescents’ SWFoL	0.67–0.89	0.89	0.63	0.13 ***	0.09 **	0.18 ***	0.20 ***	0.35 ***	0.44 ***	-

Average variance extracted: AVE. The values over the diagonal indicate squared correlations (r^2^) between constructs. The values under the diagonal indicate correlations (r) between constructs. ** *p* < 0.01 *** *p* < 0.001.

## Data Availability

Data and materials are available from the corresponding author upon reasonable request due to ethical reasons.
